# Supplementary material for: “This is you teaching you:” Exploring providers’ perspectives on experiential learning and enhancing patient safety and outcomes in ketamine-assisted therapy
Source: PLoS One. 2024 Aug 29;19(8):e0306381. doi: 10.1371/journal.pone.0306381 (PMC11361647; doi:10.1371/journal.pone.0306381)
Supplement: S1 Appendix — (PDF) [file pone.0306381.s001.pdf]

Qualitative Interview Guide

Preparatory comments to interviewers: The purpose of these interviews is to clarify, complement and expand upon our understanding of KAT and clinical outcomes. The interviews will provide program therapists with an opportunity to share and reflect upon the nature and meaning of their experiences, both with their clients and their own personal experiences. The interviews also aim to elicit feedback about the program and identify opportunities to improve the delivery, safety and efficacy of the therapy.

It is important to emphasize that the overall ordering/direction of questions takes a backseat to the maintenance of good rapport and flow vital to the objectives of the qualitative interview. Depending on the interviewer's style, it would be suggested not to ask these questions word for word, but to become knowledgeable about the purpose of each question and gather that information in a way that makes sense with the flow of the interview.

Start of interview:

"This interview will cover your experience of being a therapist in the ketamine-assisted therapy provided at Numinus clinics. It will last about 60-90 minutes. You are welcome to take breaks whenever you like. If you feel upset at any point we can stop. Do you have any questions before we start?"

Questions 1- 6: Prior Experience, Motivations & Intentions

1. Can you tell me a bit about your background and your training? {Probe: education and therapeutic experience]
    a. Did you have previous training in psychedelic-assisted therapy? What about other modalities? If so, please describe.

2. What motivated your choice to work with ketamine-assisted therapy?

3. What were your expectations or intentions for your work as a KAT therapist?

    a. Did you have any fears or hesitations about getting involved?
    b. Anything you were particularly excited about?

4. Have you had any experience with non-ordinary states of consciousness that occurred without consuming a psychoactive substance? (E.g., Holotropic Breathwork, shamanic drumming, sweat lodge ceremony, meditation retreat, etc.)
    a. What modality allowed you to enter the ordinary state of consciousness and what was your experience like?

5. If you feel comfortable sharing, can you tell me about any personal experiences you've had with ketamine or other psychedelic substances? [Remind participant that this interview is completely confidential.]
    a. If yes, probe: type of psychedelics/experiences; doses; setting/context of use (formal/ceremonial or informal)
    b. If no: What were your reasons for never trying ketamine/psychedelics?

6. Do you feel that therapists should have first-hand experience with psychedelics to do psychedelic therapy? Why or why not? [Probe: strengths and limitations of having or not having personal/direct experience.]
    a. If relevant, could you share how your own personal experiences with psychedelics inform your work?
    b. If you've had any experiential training (direct experience) with ketamine or other psychedelics, could you describe how this has impacted your training and outcomes for clients?

Questions 7-12: Therapist Experience, Personal and Professional Impacts

7. What aspects of preparing for KAT sessions did you find most important for you or your clients, and why? [Probe/examples: reading materials, podcasts, therapy manual, talking to colleagues, setting-up treatment room, i.e., music, video recording, furniture placement, lighting, blankets/pillows).
    a. Could you tell me about any preparation sessions your clients had?
    b. Was there anything you felt would have better prepared you or your clients either logistically or emotionally or otherwise? [Probe: feedback on number of sessions]

8. Please describe any impacts on your personal life or new insights about yourself after the ketamine sessions. [Probe: relationship to self, others, sense of connectedness to nature, spirituality.]

9. Please describe any professional changes in yourself since being a KAT therapist. Any professional insights or new understandings about yourself as a therapist? [Probe: any changes to your understanding of the role of the therapist, attitude toward your career, overall satisfaction with career?]

10. Did you notice any changes in your professional therapeutic approach since being a KAT therapist?

    a. Any changes to how you might prepare before sessions?
    b. Any changes in your capacity for self-care or to hold work-related stress? [Probe: What self-care practice did you use; were they effective?]
    c. Any changes to your level of emotional exhaustion?
    d. Any changes in empathy towards your clients?

11. Could you please tell me about your experience with the professional training required for this program? [Probe: did it appropriately prepared you to be a program therapist?]
    a. What was the most impactful piece of the training for you?
    b. What do you feel could have been done better?
    c. Are there any additional components of training that you would suggest future program therapists receive? Any components of training you feel future program therapists do not need to receive?

12. Do you plan to continue delivering ketamine-assisted therapy? Why/why not? [Probe: with ketamine and/or another medicine?]
    a. What do you see as the advantages or disadvantages of using ketamine as compared to other psychedelics as an adjunct to psychotherapy?

Questions 13-18: Feedback About the Program

13. How many ketamine sessions did your clients receive? How do you feel about the number of sessions? [Probe: would you have liked them to have additional or fewer sessions, why?]

14. In what form did your clients receive ketamine (sublingual, nasal spray or intramuscular)? Could you tell me a bit about how this route of administration and dose was for your clients? [Probe: was it adequate/too much or too little]

15. How many integration sessions did your clients have? Could you tell me about what your experience of these sessions were like? [Probe: anything that would have improved integration (e.g., number of sessions)]

16. Can you describe how you think KAT works and what happens to clients receiving KAT?

    a. What aspects of the program do you think were most impactful/important for clients in their healing process?
    b. What aspects do you think were most challenging for clients?

17. Please describe your overall experience being a KAT therapist in the program.
    a. What do you like about doing this work? What parts of the program felt most impactful for you?
    b. What aspects of this work do you find challenging?

18. Is there anything in the program that you wish could be different? If you could go back and re-design the KAT program, what would you do differently?

ANY FINAL COMMENTS: Is there anything else you would like to share about your experiences?
